# Anti-Inflammatory Substances in Wheat Malt Inducing Antisecretory Factor

**DOI:** 10.1007/s11130-019-00767-1

**Published:** 2019-08-21

**Authors:** E. Johansson, S. Lange, M. Oshalim, I. Lönnroth

**Affiliations:** 1 Department of Infectious Diseases, Institute of Biomedicine, Sahlgrenska Academy, University of Gothenburg, P.O.B 420, S-40530 Gothenburg, Sweden; 2 Department of Clinical Microbiology, Sahlgrenska University Hospital, Region Västra Götaland, P.O.B 7193, S-40234 Gothenburg, Sweden

**Keywords:** Wheat, Anti-inflammation, Antisecretory factor, Proteasome, TRPV1

## Abstract

Extensively malted cereals counteract enterotoxic diarrhea and inflammatory bowel diseases. This effect depends on a protein called antisecretory factor (AF), which is secreted into the blood as a larger complex known as the compleasome. In this study, we identified anti-inflammatory substances in malt and assayed their capacity to induce AF. Guaiacol and quercetin inhibited inflammation in a mouse footpad model, while catechin, sinapic acid, ferulic acid, and quercetin inhibited nitric oxide formation in RAW 264.7 cells. The proteasome activity in these cells was inhibited by vanillic acid and quercetin but not by the other tested phenols. As the transient receptor potential vanilloid 1 (TRPV1) might be involved in AF induction, the TRPV1 antagonist capsazepine was tested and shown to inhibit inflammation in mouse paw and nitric oxide formation. Catechin, ferulic acid, and sinapic acid induced AF in rat blood, and these substances were all increased in malt compared to control wheat. These phenols might therefore be of particular importance for the beneficial effect of malted cereals on inflammatory diseases. Our results further suggest that TRPV1 might play a role in the anti-inflammatory activity of phenols via the induction of AF.

**Electronic supplementary material:**

The online version of this article (10.1007/s11130-019-00767-1) contains supplementary material, which is available to authorized users.

## Introduction

Wheat and other cereal grains contain both inflammatory substances such as agglutinins and anti-inflammatory substances such as antioxidants [1–3]. Malting increases the levels of anti-inflammatory substances in cereals by releasing these substances from larger complexes [4–6], and so intake of extensively malted oats and wheat can counteract enterotoxic diarrhea and inflammatory bowel disease such as ulcerous colitis and Crohn’s disease [7–9]. The active phytochemicals seem to be certain phenols which induce an antisecretory factor (AF) in the blood [10]. AF exists both as a single protein and in a complex with proteasomes and complement factors, known as a compleasome [11, 12]. In the compleasome, the active AF sequence is exposed, regulating fluid transport in the gut and other organs [13, 14]. The aim of the present study was to elucidate the molecular background of the anti-inflammatory effect of malted cereals. Malt and individual phenols were tested for inhibition of granulocyte-mediated edema in mouse footpad, decrease in lipopolysaccharide-induced nitric oxide (NO), and inhibition of proteasome activity. The concentrations of phenols in leachate of malt and control wheat and their capacity to induce AF in rat blood were also determined.

## Material and Methods

### Test Compounds

Griess reagent, lipopolysaccharide from *Escherichia coli* O111:B4, Folin-Ciocalteu phenol reagent, and the various phenols (vanillic acid, ferulic acid, sinapic acid, catechin, quercetin, capsazepine, and N-oleoyldopamine [OLDA]) were obtained from Sigma-Aldrich. The Proteasome-Glo chymotrypsin-like cell based assay was obtained from Promega (www.promega.com). Monoclonal IgM antibodies against AF/RPN10 were produced as previously described [15]. Polyclonal antibodies against complement factor C3 were obtained from Dako (www.dako.com, item A0062). Secondary antibodies, alkaline phosphatase conjugated goat anti-rabbit IgG, and goat anti-mouse IgM were obtained from Jackson ImmunoResearch, and the solvents for HPLC chromatography were obtained from Merck.

### Preparation of Cereal Leachate and Phenols

Kossack (WW 27084) wheat was processed in a micro malting facility (Danbrew Ltd) as previously described [10]. The malted Kossack wheat and the unprocessed control wheat were extracted with boiling water and administrated to the animals as drinks (Supplementary Material).

### Animal Experiments

All experimental procedures with the mice and rats were approved by the Regional Animal Experiments Ethics Committee and conformed to EU Directive 86/609/EEC. Male DBA/1 mice and Sprague-Dawley rats were housed in a controlled environment with a 12 h light cycle. The animals had free access to water and pelleted food before and during the experimental period.

- Male DBA/1 mice (*n* = 6/group) from Møllegaard Breeding Laboratories (Lille Skensved, Denmark) were used for induction and assessment of granulocyte mediated, olive oil-triggered inflammation in the hind foot [16]. The mice were given malt leachate or 5 μM of pure phenols in drinking water as described above. After five days of drinking, 10 μl of olive oil was injected intradermally under isoflurane anesthesia on the dorsal part of the left hind paw. The mice were terminated, also under isoflurane anesthesia, 24 h after this olive oil injection, and the thickness of the footpad was measured using an Oditest spring caliper. The right paw served as a control, and the thickness of the left minus the right paw was used as an estimate of the induced footpad edema.
- Male Sprague-Dawley rats (*n* = 5/group) of body weight 250 ± 20 g (B&K AB, Stockholm, Sweden) were used for induction and estimation of compleasomes in the blood. Rats were given leachate from malted wheat or pure phenols in drinking water as described above [10]. After 14 days, the rats were terminated under isoflurane anesthesia and blood samples were drawn by heart puncture as previously described [10].

### Immunoassay

The antisecretory activity in the plasma samples was determined by performing a sandwich enzyme-linked immunosorbent assay (ELISA) as previously described [12]. A monoclonal antibody (mab) against AF was used as catching antibody and a polyclonal antibody against complement factor 3c as detecting antibody. The net absorbance at 405 nm was determined after development by a secondary antibody coupled to alkaline phosphate.

#### Cell Tests

- RAW 264.7 cells were cultured in DMEM 41965–0399 high glucose medium from Gibco supplemented with 10% fetal calf serum at 37 °C in a humidified 5% CO_2_/95% air atmosphere.
- Proteasome activity was assayed via the Glo chymotrypsin-like activity (Promega, Madison, WI, USA). In a black plate with 96 wells of 100 μl each, 10^5^ cells *per* well were allowed to adhere for 2 h and then 10 μl of phenols, 10–100 μM dissolved in dimethyl sulfoxide, was added [17]. The mixtures were incubated for 60 min at 37 °C in a 5% CO_2_/95% air atmosphere, and then allowed to equilibrate to room temperature for 20 min before 100 μl of the Proteasome-Glo reagent was added. The plates were kept in the dark for 30 min before being read in a luminescent plate reader (CLARIOstar, BMG Labtech).
- NO production was assayed by use of Griess reagent after challenge with lipopolysaccharide [17]. In a 24-well tissue culture plate, 10^6^ cells/500 μl were allowed to adhere for 2 h, whereafter lipopolysaccharide (10 ng/well) and 10 μl of phenols (50–500 μM in dimethyl sulfoxide) was added. After incubation for 24 h at 37 °C in 5% CO_2_/95% air, the supernatants were collected and stored at −20 °C. The levels of NO were assayed by adding 100 μl of Griess reagent to 100 μl cell medium and reading at 570 nm in a plate reader (VersaMax, Nordic Biolabs).

### Quantitative Assay of Phenols

The quantitative assay of catechin, ferulic, sinapic, and vanillic acids in the wheat leachate was performed at the Swedish Metabolomics Center, Swedish University of Agricultural Sciences, Umeå. The sterile filtrated leachate was applied on a HPLC HSS T3 (2.1 × 100 mm, Waters) with C13-labelled ferulic and vanillic acid as internal standards, eluted with a linear gradient of 0–100% water/acetonitrile with 0.1% formic acid, and analyzed in an Agilent 6490 triple quadrupole mass spectrometer (Hugin).

The concentration of total phenols in malt and control wheat leachate was estimated with Folin-Ciocalteu phenol reagent [18] using ferulic acid as standard.

### Statistics

Graphs were constructed using Excel 2010. A one-way Student’s t test was used for comparing mean values in order to calculate *p*-values. Statistics are presented as mean ± standard error of the mean unless otherwise stated.

## Results and Discussion

### Inflammation in Footpad

The anti-inflammatory capacity of wheat malt and various phenols was tested in mouse paw, an established animal model for inflammation [2, 16]. Inflammatory edema in the footpad was triggered by olive oil, which induces a strong granulocyte mediated T cell independent response. It has earlier been shown that phenol extracts from various plants inhibit footpad edema, but no systematic assay of the effect of specific phenols has been published.

When the mice were allowed to drink leachate from wheat malt for five days, edema was inhibited by 44%, while leachate from untreated wheat did not produce inhibition (Table 1). This is consistent with previous trials in humans showing that malted but not control cereals had an anti-inflammatory effect on the intestine [7]. We also tested individual malt phenols for their anti-inflammatory ability. Guaiacol, previously shown to have anti-secretory ability, was also anti-inflammatory (Table 1). Surprisingly, vanillic and ferulic acid had no effect on the mouse paw model, despite having previously been shown to have antisecretory effect [10]. Sinapic acid also had no significant effect in the mouse paw model. Catechin had a modest effect in the mouse paw model, while the structurally similar quercetin had a pronounced effect. Quercetin has previously been used as an anti-inflammatory agent [19] and it also exerts an antisecretory effect in the gut [10].

**Table 1:**
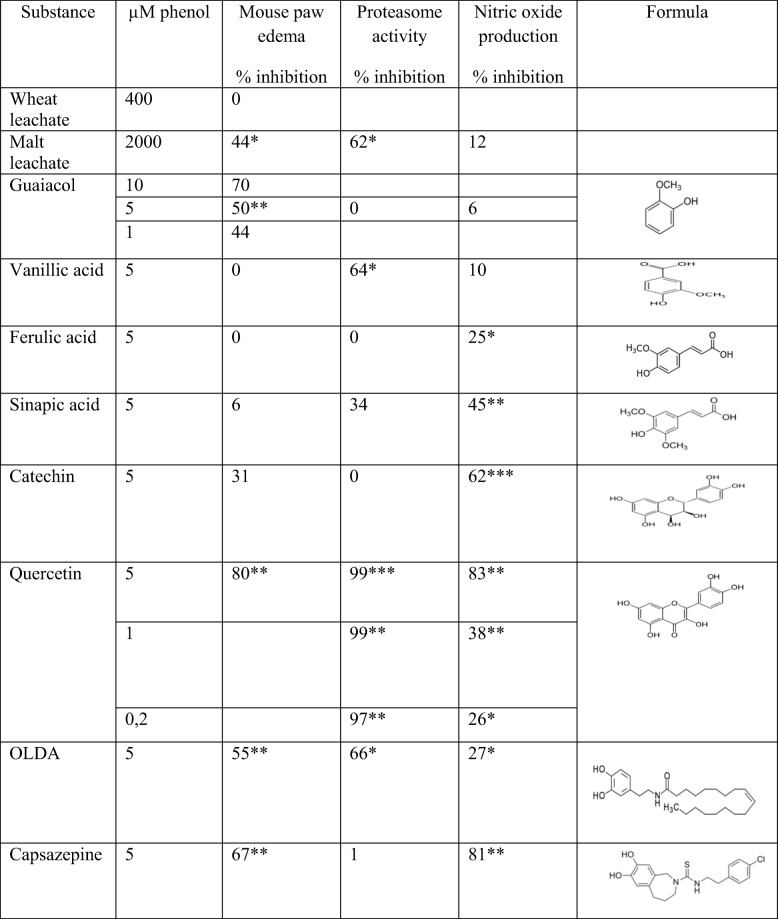
Effects of leachate and phenols on inflammation. Mice were given malt leachate or 5 μM of pure phenols in drinking water for five days, and then edema was induced by olive oil in the left hind paw, with the right paw serving as a control (*n* = 6). Effects on proteasome activity and nitric oxide production were measured in cultured RAW 264.7 cells (*n* = 6)

* *p* < 0.05; ** *p* < 0.01; *** *p* < 0.001

Since the TRPV1 receptor antagonists inhibit intestinal secretion [10], the specific agonist OLDA and antagonist capsazepine were tested in the mouse paw model. Both gave a strong inhibition which suggests that the classical TRPV1 receptor in nerves is not involved but rather a somewhat different receptor in granulocytes which probably mediate the foot edema [16].

### Cell Test

To find out more about the underlying mechanism of action of the anti-inflammatory substances, experiments were performed on RAW 264.7 cells, testing the ability to inhibit the chymotrypsin activity of the proteasomes and the ability to inhibit the release of LPS-induced NO.

The proteasome affects inflammation by its effect on nuclear factor kappa B [20], and is also involved in the induction of AF [12]. The present results showed that leachate from the malt gave a 62% inhibition of proteasome activity (Table 1). When we tested the individual phenols, vanillic acid, quercetin, and OLDA showed significant effects while guaiacol, ferulic acid, catechin, and capsazepine had no effect. The activity of quercetin was particularly pronounced, and it is therefore surprising that the structurally similar catechin had no effect. It is also noteworthy that the TRPV1 agonist OLDA and the antagonist capsazepine differed in activity. It is possible that the relatively lipophilic structures of quercetin and OLDA allowed them to penetrate through the cell wall more easily than catechin and capsazepine.

NO regulates vasodilation and is an important part of the inflammatory reaction [21]. Leachate from wheat malt produced no significant effect on lipopolysaccharide-induced NO formation, but all the individual phenols except vanillic acid gave a significant effect (Table 1). Naturally occurring proteasome inhibitors have previously been reported to inhibit inducible NO synthase [17], but this correlation was not seen in our study.

### Concentration of Active Phenols in Leachate

The level of some of the active phenols in leachate was determined by mass spectrometry using the isotope labeled substances as references. Catechin, ferulic, and sinapic acid increased 40-, 3-, and 5-fold, respectively, in malt compared to control wheat, while the level of vanillic acid was fairly constant (Table 2). Total phenol concentration increased 5-fold. This shows that malt indeed releases phenols with pronounced anti-inflammatory effect.

**Table 2:** Concentration of phenols in wheat and wheat malt

	Catechin pg/μl	Ferulic acid pg/μl	Sinapic acid pg/μl	Vanillic acid pg/μl	Total phenols mM
Wheat	8	562	40	442	0,4
Wheat malt	306	1792	195	515	2,0

### Concentrations of Anti-Secretory Factor in Blood

**Fig. 1:**
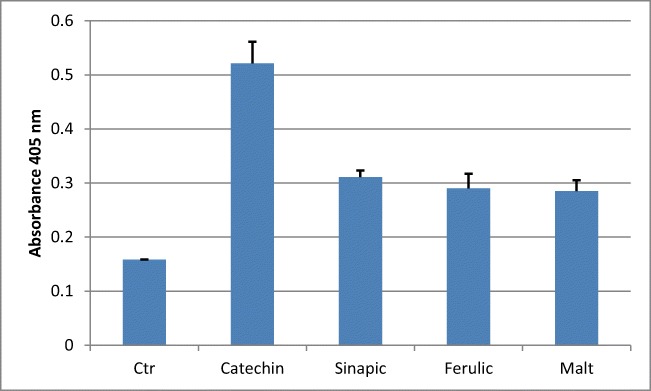
Induction of AF activity in rat blood plasma. Rats (*n* = 5/group) were given 5 μM catechin, ferulic acid, sinapic acid, or malt in drinking water for 14 days. The diagram shows the AF activity in blood measured in ELISA. Catechin (*p* < 0.001), sinapic acid (*p* < 0.01), ferulic acid (*p* < 0.05), and malt (*p* < 0.01) all produced a significant increase in compleasome concentration in relation to the control

Since previous publications have shown that AF inhibits intestinal inflammation [7], we examined whether the phenols that were increased in the malt leachate could induce AF in the blood (Fig. 1). After the rats drank low concentrations of the substances for 14 days, AF was determined in plasma by ELISA test. Catechin, sinapic, and ferulic acid induced the AF compleasome, as did wheat malt, while leachate from control wheat did not (not shown). Taking into account the concentration of the various phenols, one would expect the anti-inflammatory response to ferulic acid in the leachate to be dominant, with catechin and sinapic acid contributing only a minor proportion (Fig. 1).

## Conclusion

According to the mouse paw model, leachate from wheat malt has anti-inflammatory ability whereas leachate from unmalted wheat does not. Of the individual phenols in wheat, both guaiacol and quercetin showed anti-inflammatory effects in mouse paw. Catechin, sinapic acid, ferulic acid, and quercetin inhibited NO formation while guaiacol and vanillic acid did not. The concentrations of catechin, ferulic acid, and sinapic acid were significantly higher in the malt than in the untreated control, but there were no differences in levels of vanillic acid. Catechin, sinapic acid, ferulic acid, and malt all showed an ability to induce AF, and so might be of particular importance for the beneficial effect of malt on inflammation. Our results also suggest that inhibition of NO and the TRPV1 receptor might play a role in anti-inflammation, as previously shown for its antisecretory effect. We conclude that the inhibitory effect of malt on inflammation is exerted by phenols acting via AF.

## Electronic Supplementary Material

ESM 1 (DOCX 13 kb)

## Abbreviations

AF: Antisecretory factor
ELISA: Enzyme-linked immunosorbent assay
Ig: Immunoglobulin
NO: Nitric oxide
OLDA: *N*-oleoyldopamine
TRPV1: Transient receptor potential vanilloid 1
