# Supplementary material for: Anti-Inflammatory Substances in Wheat Malt Inducing Antisecretory Factor
Source: Plant Foods Hum Nutr. 2019 Aug 21;74(4):489–94. doi: 10.1007/s11130-019-00767-1 (PMC6900268; doi:10.1007/s11130-019-00767-1)
Supplement: Supplementary file 1 — (DOCX 13 kb) [file 11130_2019_767_MOESM1_ESM.docx]

The malted Kossack wheat and the unprocessed control wheat were milled to flour, of which 200 g was put in a glass flask and then successively mixed with 800 ml boiling water. After 3 hours of slow cooling to room temperature, the soak-water was filtered through a nylon filter and heated in boiling water for 1 hour. After cooling, the leachate was centrifuged for 30 min at 12000 × g and the supernatant frozen in small portions. The portions were thawed and diluted 1:6 with water each day during the experiment. The pure phenols were kept frozen at 5°C in 50% ethanol, and diluted in the drinking water to a final concentration of 5 µM. The drinks or the water control were administrated to the animals ad lib over 1–14 days, whereafter antisecretory activity was tested in plasma.
